# Overexpression of CD97 Confers an Invasive Phenotype in Glioblastoma Cells and Is Associated with Decreased Survival of Glioblastoma Patients

**DOI:** 10.1371/journal.pone.0062765

**Published:** 2013-04-26

**Authors:** Michael Safaee, Aaron J. Clark, Michael C. Oh, Michael E. Ivan, Orin Bloch, Gurvinder Kaur, Matthew Z. Sun, Joseph M. Kim, Taemin Oh, Mitchel S. Berger, Andrew T. Parsa

**Affiliations:** Department of Neurological Surgery, University of California San Francisco, San Francisco, California, United States of America; Faculdade de Medicina, Universidade de São Paulo, Brazil

## Abstract

Mechanisms of invasion in glioblastoma (GBM) relate to differential expression of proteins conferring increased motility and penetration of the extracellular matrix. CD97 is a member of the epidermal growth factor seven-span transmembrane family of adhesion G-protein coupled receptors. These proteins facilitate mobility of leukocytes into tissue. In this study we show that CD97 is expressed in glioma, has functional effects on invasion, and is associated with poor overall survival. Glioma cell lines and low passage primary cultures were analyzed. Functional significance was assessed by transient knockdown using siRNA targeting CD97 or a non-target control sequence. Invasion was assessed 48 hours after siRNA-mediated knockdown using a Matrigel-coated invasion chamber. Migration was quantified using a scratch assay over 12 hours. Proliferation was measured 24 and 48 hours after confirmed protein knockdown. GBM cell lines and primary cultures were found to express CD97. Knockdown of CD97 decreased invasion and migration in GBM cell lines, with no difference in proliferation. Gene-expression based Kaplan-Meier analysis was performed using The Cancer Genome Atlas, demonstrating an inverse relationship between CD97 expression and survival. GBMs expressing high levels of CD97 were associated with decreased survival compared to those with low CD97 (p = 0.007). CD97 promotes invasion and migration in GBM, but has no effect on tumor proliferation. This phenotype may explain the discrepancy in survival between high and low CD97-expressing tumors. This data provides impetus for further studies to determine its viability as a therapeutic target in the treatment of GBM.

## Introduction

Glioblastoma (GBM) is the most common and aggressive primary brain tumor with a median survival of less than two years [1], [2]. The invasive nature of gliomas is a major factor limiting complete removal despite aggressive surgical resection. Intracranial dissemination, either at diagnosis or progression, is a poor prognostic factor associated with decreased survival [3]. Despite decades of research, the mechanisms underlying GBM invasion remain to be fully elucidated. As our ability to characterize the molecular signatures of GBM improves, there is a growing need to identify markers that can predict aggressiveness and promote the development of targeted therapies. Since invasive tumors are known to confer a worse prognosis, there is a particular need to identify mediators of tumor invasion.

The epidermal growth factor seven-span transmembrane (EGF-TM7) family of adhesion G-protein coupled receptors (GPCRs) consists of proteins that are expressed mainly on the surface of leukocytes [4]. Six members of this family have been identified: CD97 [5], [6], EGF-like module containing mucin-like receptor 2 (EMR2) [7], EMR3 [8], EMR4 [9], [10], and EGF-TM7-latrophilin-related protein (ETL) [11]. CD97 has the broadest expression pattern among all members; it is found on lymphocytes, monocytes, macrophages, dendritic cells, granulocytes, and smooth muscle [12]. It is rapidly upregulated during activation of lymphocytes [12] and has been implicated in cell adhesion and migration via interactions with cell surface proteins and components of the extracellular matrix (ECM). CD97 has three known ligands: CD55, a negative regulator of the complement cascade [13], chondroitin sulfate, a component of the ECM [14]–[16], and the integrin α5β1 [17]. The association with integrins is particularly noteworthy since they have been shown to mediate invasion, migration, and angiogenesis in GBM [18], [19]. Additionally, proliferating endothelial cells in the brain are known to express chondroitin sulfate, suggesting a potential interaction between tumor and nascent vessels [20].

CD97 is expressed in thyroid, gastric, esophageal, pancreatic, and colorectal cancers [21]–[23]. It has been shown to correlate with lymph node invasion in thyroid cancer [22] and poor clinical stage in colorectal cancer [21]. Within tumors, expression of CD97 is generally highest at the invasive front or leading edge [21], [23], [24]. Functionally, CD97 has been shown to confer an invasive phenotype and stimulate angiogenesis [17], [25]. CD97 was recently implicated in GBM after suppression of Wilms tumor 1 (WT1) resulted in downregulation of the CD97 gene product [26]. Two other members of the EGF-TM7 family, EMR2 and EMR3, have also been identified in GBM, adding to evidence that this family may play an important role in glioma biology [27], [28]. We present data characterizing the expression and function of CD97 in human GBM. Using siRNA knockdown, we show that CD97 confers in invasive and migratory phenotype, but has no effect on cell proliferation. Using gene expression-based Kaplan-Meier analysis, we show that CD97 confers a poor prognosis in GBM and is associated with decreased survival. These findings provide impetus to further characterize the role of CD97 in glioma and determine its utility as a potential therapeutic target.

## Methods

### Cell Culture

Primary glioblastoma cell lines were established from surgical specimens acquired at our institution from 2 surgeons (ATP, MSB). All research activities were approved by the University of California, San Francisco Committee on Human Research (UCSF CHR), our institutional review board for human research, with both written and verbal consent provided from patients. Freshly resected tumors, pathologically confirmed as GBM, were minced with a sterile scalpel and chemically dissociated in Papain at 20 units/ml (Worthington Biochemical Corporation) for 20 minutes at 37°C. The dissociated tissue was filtered through a 70 µM cell strainer and the remaining cell suspension plated in RPMI-1640 media with 2 g/L glucose, 0.3 g/L L-glutamine, 2 g/L NaHCO_3_, 25 mM HEPES, 10% fetal bovine serum (FBS), 1% non-essential amino acids, 1% sodium pyruvate, and 1% penicillin/streptomycin. The human glioma cell lines U251, U87MG, and G55 were provided by Dr. Russell Pieper at UCSF (originally from American Type Culture Collection). These cells were grown in Dulbecco’s Modified Essential Media (DMEM) with 4.5 g/L glucose, 0.584 g/L L-glutamine, 0.11 g/L sodium pyruvate, 3.7 g/L NaHCO_3_, 10% FBS, and 1% penicillin/streptomycin.

### Immunoblotting

Cell lysates were generated by transferring 5×10^5^ cells to a 10 cm plate and observing them until they reached 75–80% confluence. Plates were washed with cold phosphate-buffered saline (PBS) and treated with 150 µl of 10× lysis buffer (Cell Signaling Technology) containing 20 mM Tris-HCl (pH 7.5), 150 mM NaCl, 1 mM Na_2_EDTA, 1 mM EGTA, 1% Triton, 2.5 mM sodium pyrophosphate, 1 mM beta-glycerophosphate, 1 mM Na_3_VO_4_, and 1 µg/ml leupeptin. Protease inhibitor tablets (Roche), phosphatase inhibitor tables (Roche), and 1 mM phenylmethasnesulfonylfluoride (PMSF) were added to the lysis buffer before use. Cells were scraped off the plate and placed on rotating chamber for 4 hours at 4°C. Raw lysates were cleared of insoluble materials by centrifugation at 13,000 rpm for 20 minutes. Protein concentrations were normalized by bicinchoninic acid (BCA) assay (Thermo Scientific) to a concentration of 1 µg/µl in 1X SDS loading buffer (10% sodium dodecyl sulfate, 1% β-mercaptoethanol, 20% glycerol, 0.2 mM Tris (pH 6.8), 0.05% bromophenol blue). Protein samples were heated to 90°C for 5 minutes then electrophoresed through a 4–20% Tris-Glycine gel (Invitrogen). Resolved proteins were then transferred to an Immun-Blot PVDF membrane BioRad), blocked in 5% nonfat dry milk (BioRad) in Tris-buffered saline with 0.05% Tween-20 (TBST), and incubated in primary antibody overnight at 4°C. Anti-CD97 antibody was used at a concentration of 1∶500 (Abcam, ab108368) and anti-GAPDH antibody was used at 1∶10,000 (Cell Signaling, #2118). Membranes were washed in TBST then incubated with secondary antibody conjugated to horseradish peroxidase (Cell Signaling) diluted to 1∶2,000 in 5% milk. Membranes were then washed and developed using the Amersham ECL Western blotting detection system (GE Healthcare).

### Immunocytochemistry

Cells were plated onto 12 mm cover glass at a density of 2.5×10^5^ cells/cover glass (Fisher Scientific), then cultured for 2 days until reaching 80%–90% confluence. After a brief wash with Dulbecco’s modified PBS (DPBS), cells were fixed with 4% formaldehyde for 5 minutes at room temperature. Cells were permeabilized with cold methanol (−20°C), then blocked in DPBS containing 5% FBS, 2 mg/ml BSA, and 0.1% Triton X-100 for 1 hour at room temperature. Slides were incubated with a 1∶100 dilution of rabbit anti-CD97 (Abcam, ab108368) or rabbit anti-α–tubulin antibody (Cell Signaling, #2125S) for 1 hour at room temperature, followed by incubation with a 1∶250 dilution of goat anti-rabbit IgG conjugated to Alexa Fluor® 488 (Invitrogen) for 1 hour. After 5 washes with DPBS, specimens were mounted on slides using a DAPI-containing mounting medium (Vector Labs). Confocal images were generated on a Zeiss LSM 510 META laser-scanning microscope.

### Small Interfering RNA (siRNA) Knockdown

To determine the function of CD97 in GBM, protein levels were transiently suppressed using small interfering RNA (siRNA). Cells were trypsinized and transferred to 6-well plates at 1×10^5^ cells per well. Transfection complexes were generated using the RNAiMax transfection agent (Life Technologies) per the manufacturer’s protocol. Silencer® select siRNA (Life Technologies, #4390824) was targeted against CD97 along with a negative control sequence (#4404020). Transfection agent and siRNA were diluted in Opti-MEM® reduced serum media (Life Technologies). Protein levels were assessed at 48 hours after transfection to confirm knockdown.

### Invasion Assay

To assess the invasive capacity of transfected cells, we utilized BD BioCoat™ Matrigel™ invasion chambers (BD Biosciences). Cells were transfected with siRNA 48 hours prior to invasion assay. Cells were transferred to the invasion chamber using the Cellstripper™ non-enzymatic cell dissociation solution (Corning). Prior to use, chambers were rehydrated with DMEM for 2 hours at 37°C then plated with 5×10^4^ cells per well. After 12 hours of migration towards a 5% FBS gradient, invasion chambers were fixed in 4% paraformaldehyde for 15 minutes, stained with crystal violet dye, and washed in PBS. Cells were counted using the AMG EVOS XL microscope.

### Proliferation Assay

After 48 hours of siRNA treatment, transfected cells were transferred to a 96 well plate at 30,000 cells per well. Proliferation was assessed using the ATPlite Luminescence ATP Detection Assay System (PerkinElmer) at 24 and 48 hours. All conditions and experiments were repeated in triplicate. All results were verified using a hemocytometer to determine cell count.

### Migration (Scratch) Assay

Cells were plated and transfected with siRNA 48 hours prior to beginning the assay. A P10 pipet tip was used to create a “scratch” in the cell monolayer. After removing debris and adding fresh media, cells were photographed every 15 minutes for 12 hours using the Zeiss AxioVision Workstation. The leading edge of migrating cells was approximated after 12 hours using ImageJ (National Institutes of Health) to generate a relative migration rate for each treatment condition.

### Analysis of TCGA Data

Analysis of 212 glioblastoma patients from The Cancer Genome Atlas (TCGA) was performed through the Cancer Molecular Atlas Portal on June 16, 2012. Gene-based Kaplan-Meier analysis was performed to assess overall survival among patients with upregulated (greater than 2-fold increase) or downregulated (less than 2-fold decrease) levels of CD97 expression compared to the mean expression level for the total patient population.

### Statistical Analysis

Statistical analysis was performed using SPSS version 20 (IBM). Kaplan-Meier plots were used to generate survival estimates with differences evaluated by log-rank test. Results of the migration, invasion, and proliferation assays were compared between non-target scramble and CD97 siRNA by independent samples t-test for each cell line. Statistical significance was defined as p<0.05.

## Results

### CD97 is Expressed in Primary Glioblastoma Cultures and Glioblastoma Cell Lines

**Figure 1:**
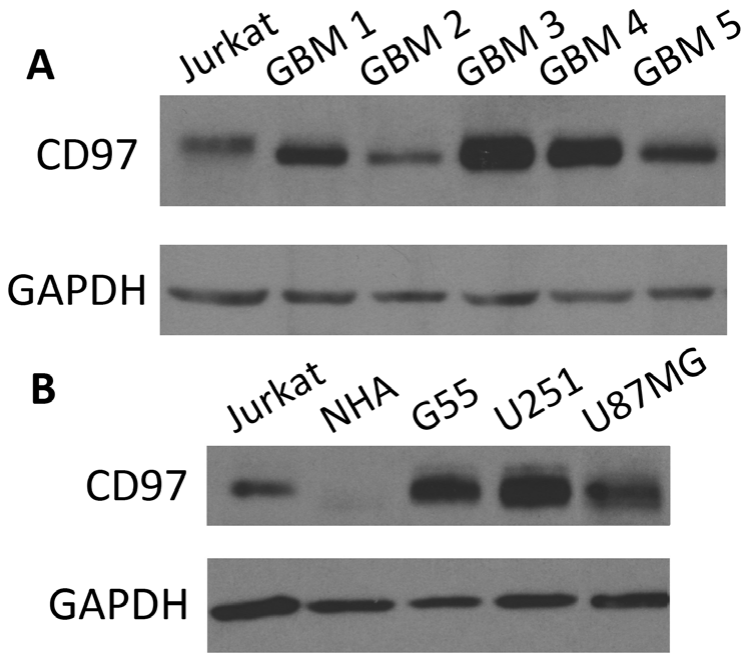
CD97 is expressed in primary GBM cultures and GBM cell lines. Five low passage primary GBM cultures and three GBM cell lines were analyzed. The five primary GBM cell lines, each isolated and cultured from a tumor resected at our institution, expressed moderate to high levels of CD97, suggesting that expression is heterogeneous among tumor specimens from different patients (A). Compared to normal human astrocytes (NHA), the GBM cell lines G55, U251, and U87MG expressed robust levels of CD97 (B). Jurkat cell lysates were used as a positive control since they are known to express CD97.

**Figure 2:**
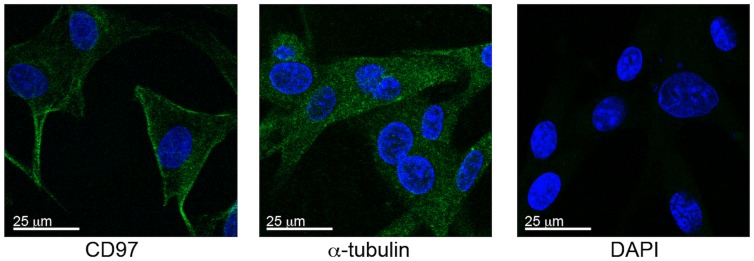
CD97 is localized to the cell membrane. To determine the distribution of CD97 within the cell we performed immunostaining on U87MG cells grown in culture. Compared to α-tubulin, a cytoskeletal marker distributed throughout the cytosolic compartment, CD97 expression was mostly localized to the cell membrane.

Given a single report demonstrating CD97 in three GBM cell lines, we sought to determine if the receptor is expressed in human GBM tissue. We collected protein lysates from five low passage primary GBM cultures generated and detected moderate to strong staining in all samples (Figure 1A). We also assessed expression in three commonly used GBM cell lines: G55, U251, and U87MG. Strong CD97 expression was detected in each cell line, but not normal human astrocytes (Figure 1B). Jurkat cell lysate was used as a positive control for CD97. To determine the distribution of CD97 within the cell, we performed immunofluorescent imaging and found that compared to the cytoskeletal protein α-tubulin, CD97 was localized to the cell membrane (Figure 2).

### CD97 Expression is Associated with Poor Prognosis

**Figure 3:**
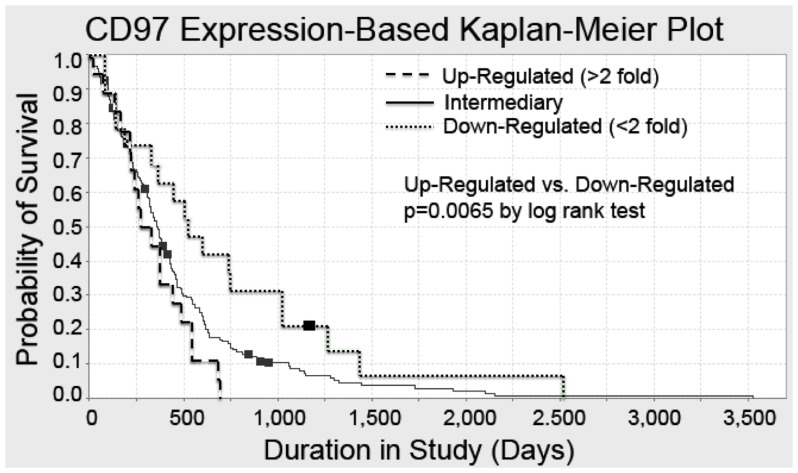
CD97 expression in GBM is inversely related to survival. Using The Cancer Genome Atlas database, we performed univariate analysis to assess a potential association between CD97 expression and overall survival. Tumors were classified as possessing intermediate expression, down-regulated expression, or up-regulated expression. Up-regulated tumors were defined as those with a greater than two-fold increase in CD97 transcript, while those with a greater than two-fold decrease were classified as down-regulated. When comparing overall survival among patients with tumors demonstrating up-regulation of CD97 to those with down-regulation of CD97, there was a significant difference in survival (p = 0.0065) demonstrating an association between increased CD97 expression and decreased survival.

Given our data demonstrating CD97 expression in GBM but not normal human astrocytes, we evaluated a large national database to investigate the association between CD97 expression and patient survival. Using The Cancer Genome Atlas (TCGA), we performed gene-based Kaplan-Meier analysis to assess overall survival among patients with upregulated (>2-fold increased expression) or downregulated (<2-fold decreased expression) levels of CD97 as compared to the mean expression level for the total patient population. A total of 212 patients were included in our analysis. Patients with upregulation of CD97 had a median survival of 250 days compared to 500 days among those with downregulated expression (Figure 3). The difference between patients with upregulated and downregulated CD97 was found to be statistically significant (p = 0.006), demonstrating an inverse relationship between CD97 expression and overall survival.

### CD97 Confers an Invasive Phenotype *in vitro*

**Figure 4:**
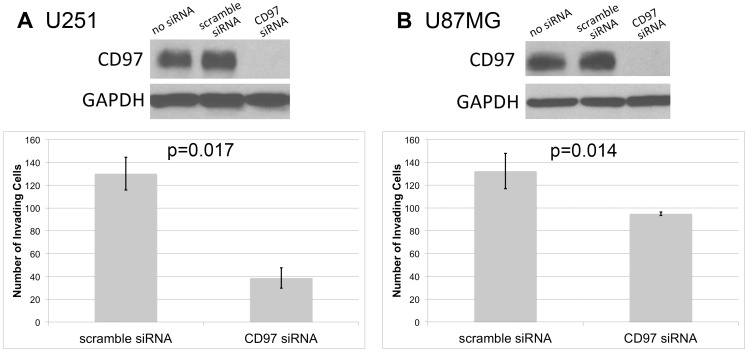
CD97 confers an invasive phenotype. U251 and U87MG cells were subjected to siRNA-mediated knockdown of CD97. In both cell lines, knockdown of CD97 resulted in decreased invasion through a Matrigel invasion chamber. In U251 and U87MG cells, invasion was decreased by 70% (p = 0.03) and 28% (p = 0.05), respectively (A, B).

To test our hypothesis that CD97 is involved in tumor invasion, we used siRNA to transiently silence gene expression and subjected cells to an invasion assay. After 48 hours of treatment with anti-CD97 siRNA or a non-target scramble siRNA, knockdown of CD97 in U251 and U87MG cells was confirmed by Western blot. We then analyzed the effect of CD97 silencing on glioma cell invasion through a Matrigel invasion chamber (Figure 4). In the U251 cell line, the number of invading cells per 10× field decreased from 130±14 in the non-target siRNA group to 39±9 in the CD97 siRNA group (p = 0.017). The number of invading U87MG cells decreased from 132±15 in the non-target scramble siRNA group to 95±1.5 in the CD97 siRNA group (p = 0.014). The relative reduction in invasion after CD97 knockdown was 70% among U251 cells and 28% among U87MG cells.

### CD97 Promotes Cell Migration *in vitro*

**Figure 5:**
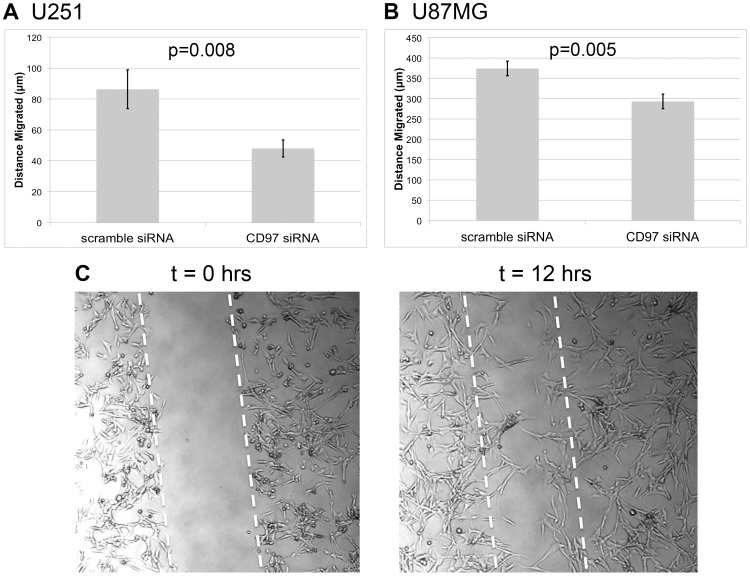
CD97 is associated with cell migration. U251 and U87MG cells were subjected to siRNA-mediated knockdown of CD97. In U251 cells, knockdown of CD97 decreased migration by 44% (p = 0.02) (A). In U87MG cells, knockdown of CD97 decreased migration by 22% (p = 0.005) (B). Migration was quantified by measuring the difference in distance between the leading edge at the initiation of the experiment and after 12 hours of incubation (C).

Since tumor invasion requires both degradation of ECM components and cell migration, we next sought to determine if CD97 was involved in migration. We assessed migratory capacity through an *in vitro* scratch assay with continuous video monitoring to calculate the distance travelled over a 12 hour period. Compared to cells treated with a scramble siRNA, those receiving CD97 siRNA travelled a shorter distance (Figure 5). U251 cells treated with a non-targeting scramble siRNA migrated 86±13 µm compared to 48±6 µm among those treated with CD97 siRNA (p = 0.008). U87MG cells treated with a non-targeting siRNA migrated 374±18 µm compared to 293±18 µm in those treated with CD97 siRNA (p = 0.005). The relative reduction in migration was 44%% in U251 and 22% in U87MG cells.

### CD97 is not Associated with Proliferation

**Figure 6:**
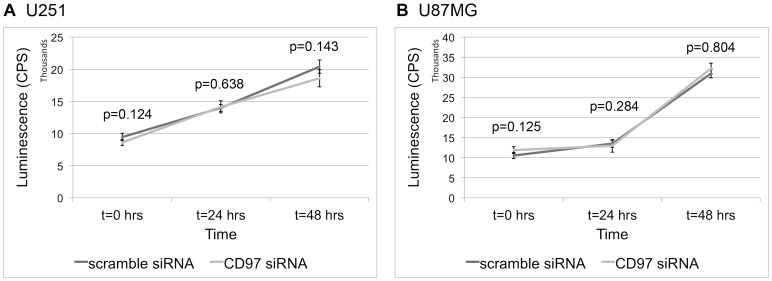
CD97 does not affect cell proliferation. U251 and U87MG cells were subjected to siRNA-mediated knockdown of CD97. Cells were quantified using an ATP-based luminescent assay, with luminescence quantified in counts per second (CPS). In both U251 (A) and U87MG (B) cells, there was no difference in proliferation at 24 and 48 hours after confirmed protein knockdown.

Cell proliferation is a fundamental component of tumorigenesis. Given the effect of CD97 on tumor migration and invasion, we sought to determine if it regulated cell proliferation. Cells were treated with siRNA against CD97 or a scramble control sequence, and proliferation was evaluated using the ATPlite Luminescence ATP Detection Assay System (PerkinElmer). At 24 and 48 hours after confirmed protein knockdown, there was no difference in the rate of cell proliferation in the CD97 siRNA treated cells compared to controls (Figure 6). These results were confirmed by repeating the experiment and using a hemocytometer to determine the cell count at 24 and 48 hours.

## Discussion

The invasive nature of GBM has been recognized for over a century. In 1934, Voris reported that nearly 25% of frontal lobe GBMs had bilateral infiltration across the corpus callosum [29]. Thirty years later, Matsukado reviewed 100 grade 3 and grade 4 astrocytomas and found that nearly 50% had bilateral extension [30]. More recently, disseminated and multifocal GBM have been shown to confer a worse prognosis [3]. Microscopic invasion makes truly complete surgical resection of GBM impossible. Although several markers of tumor proliferation and metabolism have been identified in GBM and are used clinically, prognostic markers of invasive capacity are largely lacking. Such markers can offer prognostic value and may identify new targets for therapy. In this study we demonstrate that CD97 expression has a functional effect on tumor migration and invasion *in vitro*, and correlate expression with survival in a large cohort of GBM patients. Our data suggests that CD97-mediated tumor invasion may be an important contributing factor to the observed decreased survival associated with CD97 overexpression.

CD97, along with other members of the EGF-TM7 family, possesses a unique structure involving a large extracellular domain linked to a seven-spanning transmembrane subunit. The CD97 transcript is alternatively spliced to produce isoforms with 3–5 EGF-like domains. These isoforms vary in their binding affinity and may confer functionally distinct phenotypes [17], [21], [25]. Although once thought to be leukocyte restricted, CD97 is now believed to play an important role in a number of malignancies. In thyroid cancer, CD97 overexpression is associated with increased tumor grade and is a marker of dedifferentiation [24]. In colon cancer, its expression has been demonstrated at the leading edge and correlates with poor clinical stage and increased lymphatic invasion [21]. Using an animal model of colon cancer, Becker and colleagues showed that CD97 was localized to E-cadherin-based adherens junctions and strengthened lateral cell-cell contact between enterocytes [31]. The authors also showed that overexpression of CD97 lead to upregulation of membrane-bound β-catenin, with subsequent activation of Akt. In prostate cancer, CD97 has been found in nearly 60% of clinical samples, across Gleason scores, but is not expressed in normal prostate [32]. In the same study, Ward and colleagues found that CD97 induces Gα_12/13_-dependnent RHOA activation and that depletion of CD97 reduced bony metastases by over 50%. The authors also showed that CD97 heterodimerizes and positively regulates lysophosphatidic acid receptor 1 (LPAR1) signaling, a well-established mediator of tumorigenesis and metastasis in prostate cancer [33].

Our findings in this study expand upon an initial report by Chidambaram et al. identifying CD97 expression in GBM [26]. The authors demonstrated a decrease in CD97 transcript after suppression of Wilms tumor 1 (WT1) by siRNA. The authors then identified CD97 expression in three GBM cells lines and showed that knockdown of CD97 resulted in decreased cell invasion. The findings presented in our study characterize CD97 in low passage primary GBM cultures and show that CD97 is also associated with cell migration, but not proliferation. Although the difference in migration and invasion after CD97 knockdown were not as significant in the U87MG cell line compared to U251, we believe this is attributable to differences in cell phenotype, since U251 are known to behave more aggressively and exhibit increased invasiveness *in vivo*. Most importantly, we provide the first report of an association between CD97 expression and survival in patients with GBM based on pooled analysis of patients from the TCGA. There are limitations to using TCGA data since these results do not control for known prognostic factors including age, Karnofsky Performance Status, and extent of resection. Regardless, this univariate data is compelling and provides impetus for further studies on CD97 in malignant glioma. Furthermore, different CD97 isoforms are known to confer different phenotypes in other malignancies. We are currently investigating these isoforms and their associated phenotypes.

Although the mechanisms underlying glioma invasion have not been completely characterized, several important mediators have been investigated. Glioma cells are known require certain ECM proteins in order to switch to a migratory phenotype; studies have shown that glioma cells do not move when propagated in serum free media, but become motile when treated with specific ECM components including laminin, fibronectin, and collagen [34]. Integrins have also been shown to play an important role in glioma invasion [19]. These transmembrane glycoproteins are heterodimers composed of an α and β subunit; they are capable of interacting with elements of the ECM and triggering signal transduction. The β1 subunit in particular has been shown to play an important role in malignant behavior and invasion of gliomas [35], [36]. More recently, the integrin αvβ8 has been shown to play a central role in GBM angiogenesis and perivascular tumor invasion [18]. The association with integrins and invasion is particularly noteworthy since CD97 has been shown to demonstrate strong interactions with integrin α5β1, which is expressed in GBM and other cancers [37]; coengagement of CD97 with both chondroitin sulfate, a component of the ECM, and α5β1 synergistically initiates endothelial cell invasion [17]. Integrins are being actively investigated as therapeutic targets for GBM; EMD121974 (Cilengitide), a peptide targeting the RGD-motif of integrins, showed modest efficacy in patients with recurrent GBM and will likely be subjected to future studies in combination with cytotoxic therapies [38].

Functionally, CD97 has been shown to promote cell migration and invasion, as well as increase expression and secretion of matrix metalloproteinase-2 (MMP2) and MMP9 in the fibrosarcoma cell line HT1080 [25]. By degrading components of the ECM, MMPs create physical space for glioma cells and may be critically important in promoting glioma invasion. Cathepsin B, a cysteine protease, and MMP-9 have been shown to participate in tumor growth, invasion, and angiogenesis in GBM; RNA interference was effective in suppressing this phenotype, suggesting a potential therapeutic application [39]. MMP-1 is expressed in GBM and capable of cleaving elements of the ECM or activating downstream signaling pathways that promote oncogenesis and invasion [40]. There is compelling data demonstrating the efficacy of MMP inhibitors *in vitro* and in animal models [41], [42]; they have been evaluated in clinical trials for patients with glioblastoma, but produced mixed results [43], [44]. In addition to interaction with integrins, CD97 may promote glioma cell invasion by increasing secretion of MMPs.

There are challenges to using CD97 as a therapeutic target given its expression on normal leukocytes, but several studies have made attempts to modulate or block its expression in certain pathologic conditions. Kop and colleagues used a systemically administered CD97 neutralizing antibody in a rodent model of arthritis to decrease joint inflammation and clinical signs of disease [45]. The authors found no difference in important cytokine profiles, particularly IL-10 and interferon-γ (IFN-γ), suggesting that the antibody was exerting its beneficial effect by interfering with the binding of CD97-positive leukocytes and the ligands chondroitin sulfate, CD55, or integrin α5β1. Using a thyroid cancer cell line, Park et al. showed that troglitazone, a peroxisome proliferator-activated receptor-γ (PPAR-γ) agonist, can downregulate surface expression of CD97 [46]. Sodium pyruvate has been shown to decrease CD97 expression in oral squamous cell carcinoma cell lines (OSCC) [47], while retinoid acid causes similar effects in both OSCC and thyroid cancers [48]. Taken together, these studies suggest that clinically relevant modalities can be used to modulate the expression of CD97 and that translation of these findings into *in vivo* pre-clinical models is possible. GBM is characterized by genetic heterogeneity and expression of CD97 likely varies across tumors. However, CD97 targeted therapies may hold promise as a component of molecularly targeted anti-GBM strategies. Future studies must utilize *in vivo* models to more thoroughly characterize the role of CD97 in glioma invasion. Xenograft models represent a first step towards correlating the clinical data we have presented with an observable phenotypic change *in vivo*, however given the importance of immune cells in the glioma microenvironment, an immunocompetent model is ideal. Use of a syngeneic tumor model with genetic silencing of CD97, as well as neutralizing antibodies or pharmacologic agents known to decrease CD97 expression, will further improve our understanding of CD97 in glioma invasion and potentially influence future treatment paradigms.

### Conclusions

Our data demonstrates, for the first time, an association between CD97 expression and survival in patients with glioblastoma. We believe this difference can be explained by an associated increase in tumor migration and invasion, but not proliferation. Future studies are warranted to determine if blockade or downregulation of CD97 confers a similar survival advantage *in vivo*.
